# Violence against Women by Their Intimate Partners in Shahroud in Northeastern Region of Iran

**DOI:** 10.5539/gjhs.v6n3p117

**Published:** 2014-02-27

**Authors:** Sepideh Hajian, Katayon Vakilian, Khadijeh Mirzaii Najm-abadi, Parastoo Hajian, Mehrdad Jalalian

**Affiliations:** 1 Department of Midwifery and Reproductive Health, Faculty of Nursing & Midwifery, Shahid Beheshti University of Medical Sciences, Tehran, Iran; 2 Department of Midwifery, Nursing and midwifery school, Arak University of Medical Sciences, Arak, Iran; 3 Department of Midwifery and Reproductive Health, Faculty of Nursing & Midwifery, Mashhad University of Medical Sciences, Mashhad, Iran; 4 Shohada-e-Tajrish Hospital, Faculty of Medicine, Shahid Beheshti University of Medical Sciences, Tehran, Iran; 5 Electronic Physician, Mashhad, Iran

**Keywords:** domestic violence, spouse abuse, battered women, Iran

## Abstract

**Background:**

Violence against women is one of the worst consequences of cultural, political, and socio-economic inequalities between men and women. Intimate Partner Violence (IPV) has been identified as an important cause of morbidity from multiple mental, physical, sexual, and reproductive health outcomes. Nonetheless, the prevalence and related factors of this international problem have not been investigated extensively in some parts of the world.

The aims of this research were to determine the prevalence of physical and mental violence perpetrated by men against their intimate partners and to assess the associated factors of partner violence among women in Shahroud in northeastern region of Iran in 2010.

**Methods:**

This Cross-Sectional study was conducted in Shahroud, in northeast of Iran in 2010. Cluster sampling was done from primary health service institutions, universities, public schools and governmental organizations throughout the city and six hundred married women completed the study. A structured questionnaire with 34 items was designed in three parts to assess the physically (10 items) and mentally (15 items) violent acts by a current intimate male partner and identify collative behaviors (9 items) of victims. The Logistic regression analysis was applied to determine the net effect of background variables on the IPV occurrence within the past year.

**Results:**

About 20% of the participants experienced at least one type of physical violence. Increased risk of physical violence was positively associated with the younger age of the couple (OR=3.08, P< 0.05), lower education (OR=2.28, P<0.01) and having a semi-manual skilled occupation of husband (OR=3.62, P<0.05), husband’s heavy cigarette smoking (OR=2.62, P<0.01), and his drug abuse (OR=2.1, P<0.05). About 85% of the women had experienced mental harassment within the past twelve months. Logistic Regression Analysis found that lower education (OR=3.06, P<0.01) and having semi-manual skilled occupation (OR=3.8, P<0.05) of husband, increasing years of marriage (OR=2.8, P<0.01), husband’s heavy cigarette smoking (OR=2.3, P<0.01) and his abusing the use of drugs (OR=3.4, P<0.01) had significant associations with women’s experience of mental violence.

**Conclusions:**

Some socioeconomic characteristics such as educational level, occupational status of men, heavy smoking and drug abusing are associated with the occurrence of violence against one’s intimate partner. Since IPV is usually unreported, healthcare providers should be aware of the risk factors associated with domestic violence to be able to design preventive measures against its negative health outcomes in women.

## 1. Introduction

The term “intimate partner violence” (IPV) is used to describe the brawling bawling manner that happens between family members and close relatives. This situation has been identified as an important cause of morbidity from multiple mental, physical, sexual, and reproductive health outcomes. In addition, it is associated with a range of adverse consequences that have adverse effects on the health of the victims’ children (Abramsky et al., 2011; Sadock et al., 2009). This problem is described as a shameful human rights violation, and it has short- and long-term impacts on women’s health, including unsafe abortions, fetal distress during pregnancy, pre-term labor, low weights of newborns, venereal diseases, physical disorders, tendencies toward addictions, intention to commit suicide, depression and anxiety (Ngoc Do, 2013; Rahman et al., 2012; Rodrigues et al., 2008). International Conference on Population and Development (ICPD) has emphasized to reduce violence against women as one of the priorities for the health of communities, and elimination of violence against women is a central strategy for the achievement of women’s empowerment and gender equality, which are the two main objectives of Millennium Development Goals (Elsberg, 2006).

According to WHO the global prevalence of physical and/or sexual intimate partner violence women was 30 % and the highest rates were reported from the African, Eastern Mediterranean and South-East Asia Regions (Peltzer & Pengpid, 2014). Population-based research has increased on this subject during recent years, and the prevalence of this health problem and associated factors differ globally across various populations. According to a large population-based, household survey on IPV against women conducted in 15 countries, the reported lifetime prevalence of physical or violence by a sexual partner, or both, varied from 15% to 71% (Garcia- Moreno et al., 2006). Nonetheless, the data are still limited from some regions, such as Asia - especially the Middle East - and a few studies have suggested different prevalence rates of IPV, i.e., 54% in Pakistan (Naeem et al., 2008), 23.1% in Syria (Maziak & Asfar, 2003), and 13.4% in Turkey (Toprak et al., 2009). Considering the negative consequences of this problem on the family’s health, understanding the associated factors of IPV in different populations with different cultural norms, political circumstances, and laws requires more studies in this area.

There are some reports from a few studies about IPV in Iran, which is located in the Middle East. Iran has a population of 75 million people. It is relatively culturally diverse with several ethnicities in some rural, developed, and metropolitan regions. A few studies have indicated a wide range of IPV prevalence rates in Iran. Results from research in Urmia, a northeastern province in Iran, indicated that the most prevalent form of violence was physical violence (50%), followed by mental/emotional (25.7%) and financial (23.2%). These problems were more prevalent among illiterate and young women (Arefi, 2003). However, findings from two studies from Isfahan, a large central province (Malek Afazali et al., 2004), and Khuzestan, a southwestern province (Nuh Jah et al., 2011) showed that the most common form of violence against women was the verbal-mental form (up to 64% in Isfahan and 41% in Khuzestan), while the physical, sexual, and financial forms were next in that order. Hence, these statistics cannot be applied as being representative of the overall prevalence rate in Iran, because each region of the country has its own set of cultural norms and socioeconomic status. Nonetheless, the majority of studies in this area includes the capitals of provinces in Iran and do not encompass the other sites. Hence, further population-based studies are necessary, as Iranian studies on this topic in general are still limited.

The present study was undertaken in an attempt to deal with the above-mentioned issues, i.e., the lack of clear statistics, the difficulty in reaching people in some areas of the country, the paucity of documents about IPV in various parts of Iran, and the importance of IPV to the health of women. In addition, this research was accomplished in one of the northeastern cities in Iran, Shahroud, situated in Semnan Province for two main reasons. First, there was not any population-based survey to estimate the prevalence of partner violence rate in Shahroud and any document related to IPV was gathered and referred to the forensic medicine organization of Semnan Province and final statistics used to be reported for the whole province and data was not specific for Shahroud. Second, the authors as reproductive health researchers confronted battered women attending the health centers to receive reproductive health services in several situations while there was not routine case finding program for this issue in the health facilities, so such cases are usually ignored and remained as silent victims. Indeed, prior to preventing programs and social interventions, performing preliminary study to estimate the prevalence and identify the related factors of IPV can provide clear data for this purpose. Specific objectives of the study were: a) To estimate the prevalence and intensity of two common forms of IPV, i.e., physical and mental violence by an intimate partner, and b) To determine the factors associated with the risk of first-time IPV among women in Shahroud, in north east of Iran.

## 2. Materials and Methods

### 2.1 Research design and Setting

The present cross-sectional study was accomplished for the first time in Shahroud among married women who had ever lived with an intimate partner in the past 12 months. Shahroud is a northeastern city in and capital of Shahroud County, Semnan Province, Iran. It is the largest and the most populous city of Semnan Province and its county has same position in the province. At the 2011 census, its population was 238,830, in 70,700 households (Statistical Center of Iran, 2012).

### 2.2 Sampling

#### 2.2.1 Sample Size

According to the related Iranian literature and wide range of the IPV the sample size was estimated at 645 women, considering 15% attrition (using a single cross-sectional study formula):

n= Z^2^.p. (1-p). (DEFF) / d^2^

Where: Z=1.96, p=0.7, DEFF (Estimated design effect)=2, d=0.05

As the sample size required for a cluster study is larger than simple random sampling and our study’s clusters may vary for some particular background variables, the DEFF considered around two, which means the variability is not the same as simple random sampling methods. We assumed that the greater DEFF increases the sample size and increase a desired level of precision. Although the present study was based on a relatively small sample size, 645 women, data were collected from four different directions in several and diverse sites across the city, so it seems that sample size was large enough to produce sufficient power for a number of effects to be statistically significant.

#### 2.2.2 Sampling Methods

Using the multi-stage sampling method first, the list of primary health services, universities, public schools, center of women group meetings, and governmental organizations were listed throughout the city. The reason for selecting those localities was to increase the possible access to employed and unemployed women. In addition, “out-of-home sampling” was chosen for clients and patients at hospitals and primary healthcare clinics, because the security and/or privacy of women could not be guaranteed at their homes due to the probable presence of their family members. Second, in order to do a randomized cluster sampling, the city was divided into the four portions as, north, south, west and east areas. Then, from all governmental organizations, at least one organization was selected randomly. However, according to the sensitivity of the IPV issue, two organizations did not allow the researchers for sampling. Consequently, two public schools, two public hospitals, two universities, town council, the office of public health insurance and a private center of women group meetings in the city were randomly selected. On the other part, all 10 primary health services through the city were included into the clusters and the number of sampling units chosen from each center was based on its population.

### 2.3 Measurement Tool

We designed a structured questionnaire based on WHO’s Study on Women’s Health and Domestic Violence Against Women to assess the physically and mentally violent acts by a current or former intimate male partner (Garcia-Moreno et al., 2006). Although the questionnaire was validated and used formerly in other Iranian studies (Agha Khani et al., 2002; Farrokh, Eslamloo, & Booshehri, 2007; Kazemi & Navvabi, 2004), we modified and validated our instrument using the content validity index (CVI) for each item based on literature experts and representatives of the target population (Grant & Davis, 2006; Polit & Beck, 2006). At first, the questionnaire was developed with 39 items based on the related literature in this domain. Then, researchers asked eight experts from the Departments of Reproductive Health, Midwifery, Psychiatry, Psychology, Sociology, and Epidemiology at the medical university to rate items based on their relevancy to the study’s objectives, simplicity, and clarity on a four-point scale. Then, items that had CVI values greater than 0.8 were retained, those with values in the range of 0.71 - 0.79 were returned to the researchers for reconsideration, and those that had values less of 0.70 or less were deleted. In addition, the experts were asked to suggest additional abusive items. Following this step, 20 eligible women from 10 separate households were asked to evaluate the scale for clarity, simplicity, and relevancy from the population’s perspectives. The total CVI value of the questionnaire was estimated to be 0.74, and the final instrument consisted of 34 items that were designed to assess physical and mental violence. Furthermore, we tested internal consistency (“reliability”) using Cronbach’s alpha. It is most commonly used when researchers have multiple, Likert-type questions in a questionnaire that form a scale (Gliem & Gliem, 2003). Cronbach’s alpha coefficient reached 0.92 (excellent), 0.89 (good), and 0.88 (good) for physical abuse, mental abuse, and collative behavior items, respectively.

Finally, the instrument prepared for the study consisted of the following parts: 1) general and specific demographic questions possibly associated with domestic violence, such as a history of any kind of childhood violence, the duration of the marriage, the grade given the marriage by both people involved, the number of children and their genders, the presence of children from previous marriages, and living with relatives. We used housing tenure status instead of household income, because, in some internal pilot studies, most respondents had refused to provide income information, and owning a home was strongly associated with higher income. 2) Measurements for assessing two kinds of IPV within past 12 months - physical (10 items) and mental violence (15 items) and 3) adaptive or collative behaviors of victims (9 items) to identify how women collate or cope with the problem. A five-point Likert-type response format was used to assess women’s experiences of violence in each part. Women were asked a group of questions about whether they had ever experienced any kind of physically and/or mentally violent act in the 12 months preceding the study. Women who did not report any violence on the part of their partners were categorized as ‘never abused;’ if they reported at least one violent act, they were categorized to ‘mild violence,’ and three to five violent acts were categorized as ‘moderate violence,’ and more than five violent acts were categorized as ‘severe violence.’ Every question was graded from the score of 0 for “never” to the score of 3 for “more than 5 times” for violent behavior. Hence, women who reported having experienced any act of physical and/or mental violence in the past year were categorized to ‘current violence.’

### 2.4 Data Collection

Two separate trained researchers collected data from the centers among married women with several socio-economical statuses who had ever lived with an intimate partner in the past 12 months conducted sampling. After defining the study’s objectives to the participants and obtaining their informed consent, they were assured that their verbal and written responses to the questions would be remained completely confidential. The questionnaires were anonymous and completed by participants. In case of illiteracy, trained female researchers asked the participants to answer the questions orally, and the researcher completed the questionnaire based on their responses. All interviews were conducted in a private room without the presence of a third party. During the study, women who needed counseling or treatment services were referred to the relevant sources of assistance if they asked for help. Data gathering was started on May 2010 and lasted for two months and six hundred women completed the study.

### 2.5 Ethical Considerations

Ethical approval for the study was obtained from the ethical review group and the Deputy of Research at the Shahroud University of Medical Sciences under code 8740. In addition, Informed consent was obtained from all study participants.

### 2.6 Statistical Analyses

Data were analyzed using SPSS software, version 17. Between-group differences were measured using the t- test and the one-way ANOVA test if the data had a continuous spread, and the chi-square test was used for categorical variables to test the association between the variables. We applied logistic regression to identify the factors associated with the likelihood of experiencing physical and mental violence by a spouse and by other significant partners. In case of missing data, that case was excluded from the analysis for the specific variable.

Estimation of odds ratio (OR) greater than 1 indicated as a risk factor for domestic violence, while an OR less than 1 indicated the presence of protective factors against domestic violence. However, our study had a cross-sectional design, and we analyzed the association between possible risk factors and domestic violence rather than a causality relationship. Statistical significance was considered at the 5% level.

## 3. Results

### 3.1 Participants’ Socio-Demographic Characteristics

Among the 645 participants, 600 women completed the study. The mean age of the women was 36 (±11), and about half of them had husbands who were more than five years older than they were. The mean of the years of marriage was 15 (±11), and more than half of respondents were housewives. About 20% of participants did not have any children, more than half of them had two children, and one third had a daughter and a son. Less than 5% of the respondents and their spouses had experienced second marriages and less than 3% of them lived with their children from previous marriages. Around 94% of participants lived in a nuclear family, including couple and children. Participants’ characteristics are shown in Table 1.

**Table 1:** Characteristics of participants

Characteristics	n=600	percent
**Woman’s age**		
15-25	107	17.8
26-35	178	29.7
36-45	163	27.2
≥ 46	152	25.3
**Husbands’ age**		
15-25	109	18.1
26-35	186	31.0
36-45	132	22.0
≥ 46	173	28.9
**Woman’s education (years)**		
0 to 5	95	15.8
6-12	270	45
> 12(college)	235	38.2
**Husbands’ education (years)**		
0 to 5	88	14.7
6-12	298	49.7
> 12(college)	214	35.6
**Woman’s occupation**		
housewife	311	51.7
semi-manual skilled(laborer)	17	2.8
Official employed	206	34.5
Self-employed	66	11
**Husbands’ occupation**		
Official employed	275	45.8
Self-employed	234	39.0
Manual skilled	49	8.2
semi-manual skilled(laborer)	42	7.0
**Housing tenure**		
Owner occupied	330	55.0
Rented	192	32.0
others	78	13.0
**Duration of marriage (years)**		
Up to 5	153	25.5
6-10	142	23.7
11 and more	305	50.8
Husband’s smoking (≥25 cigarettes/day)*	127	22
Husband’s drug abusing*	44	7.3
**Consanguinity marriage**	64	19.6
History of any kind of reported child violence(yes)*	43	5.7

* Some data was missing.

### 3.2 Physical Violence

The study’s findings showed that 19.6% of participants had experienced at least one kind of physical violence within the past 12 months, categorized as mild (11.4%), moderate (6.1%) and severe (2.1%) forms of physical abuse. Physically violent acts included pushing, jostling, slapping, fisting, kicking, or throwing objects at the women; however, we categorized whipping, purposeful burning, choking, and threatening with a knife to be severe abuse, even if they only occurred once.

A history of any kind of reported childhood violence in couples, gender of the children, age gap with the spouse, living with relatives (extended family versus nuclear form), housing tenure status, consanguinity marriage, duration of marriage, grade of marriage, and living with children from a previous marriage did not show any significant association with physical violence. Alternatively, the younger age of couples, primary education, being a housewife or engaging in semi-manual skilled work, heavy smoking, and drug abuse by the spouse were associated with increased risk of physical abuse in the past year compared to no reports using the Chi-square test (Table 2). All of the factors that were significant at the 5% level were included in the logistic regression analysis. We used the Enter method for these analyses. After adjusting for background variables in the model, variables, such as the age of woman, educational level of the spouse, the husband’s occupation, heavy cigarette smoking, and drug abusage of the spouse, remained significant factors in the final model associated with having experienced physical violence by the spouse. The younger age of women was strongly associated with increased risk of having experienced physical violence, since women aged 15 to 25 about three times more likely (P=0.013) to have experienced physical violence than women older than 35, and those in the age range of 26 to 35 were about 2.4 times (P=0.035) more likely to have this experience than older women. Moreover, women whose husbands’ educational levels were 0 to 6 years and 6 to 12 years were about 2.3 times (P=0.001) and 2 times (P=0.007), respectively, more likely to experience physical violence compared to those women whose husbands had higher levels of education. Also, women whose spouses were working as manual laborers and semi-manual skilled workers were 2.9 (P=0.016) and 3.6 times (P=0.03), respectively, more likely to report physical violence than women whose spouses were official employees. Women whose spouses smoked 25 or more cigarettes per day (heavy smokers) had about 2.6 times (P=0.001) the chance of experiencing physical abuse compared to women whose husbands were non-smokers. Likewise, women whose husbands were drug abusers were 2.1 times (P=0.02) more likely to report physical violence compared to those whose husbands were not drug abusers (Table 3).

**Table 2:** Descriptive data for demographic characteristics and OR and 95% CI for associations with physical and mental violence among study population (n = 600)

Variable	Physical violence OR (95% CI)	Mental violence OR (95% CI)
**Woman’s Age**		
15-25	3.14** (1.48-6.63)	1.3 (0.6-2.7)
26-35	2.23* (1.08-4.61)	2.6* (1.9-3.6)
36-45	1.98 (0.94-4.18)	2.8* (1.82-3.15)
≥ 46	Reference	Reference
**Husband’s age**		
15-25	1.80 (0.74-4.37)	1.22(0.99-3.34)
26-35	2.15** (1.25-3.70)	2.10* (1.12-4.45)
36-45	1.50 (0.82-2.73)	1.1 (0.2-2.5)
≥ 46	Reference	Reference
**Woman’s education (years)**		
0 to 5	2.46** (1.32-4.59)	1.73* (1.02-3.1)
6-12	1.97** (1.20-3.24)	1.90* (1.11-2.50)
>12 (college)	Reference	Reference
**Husband’s education**		
0 to 5	3.21** (1.55-6.64)	3.10** (1.72-5.66)
6-12	3.69*** (2.13-6.40)	3.44*** (1.98-6.60)
>12 (college)	Reference	0.6 (-0.78- 1.82)
**Woman’s occupation**		
Housewife	1.66*(1.08-2.55)	2.2* (1.09-3.6)
Semi- manual skilled (laborer)	1.80* (1.1-3.4)	1.8* (1.1-2.4)
Official employed	Reference	Reference
Self- employed	1.04(0.98-1.34)	1.6* (0.81-2.8)
**Husbands’ occupation**		
Official employed	Reference	0.7(-0.29- 2.02)
Self-employed	2.03** (1.24-3.31)	1.14 (0.50-3.11)
Manual skilled	5.83*** (2.92-11.60)	4.88*** (2.30-6.22)
semi-manual skilled(laborer)	7.15*** (2.81-18.21)	5.42*** (3.78-7.90)
**Housing tenure**		
Owner occupied	Reference	Reference
Rented	2.00* (1.27-3.17)	1.6* (0.89-5.28)
others	2.00* (1.06-3.76)	1.9*(0. 2-4.49)
**Husband’s smoking (≥25 cigarettes/day)**		
Yes	3.27*** (2.06-5.20)	3.1*** (1.23-5.60)
No	Reference	Reference
**Husband’s drug abusing**		
Yes	2.26** (1.15-4.44)	4.8*** (2.12-6.89)
No	Reference	Reference
**Duration of marriage (years)**		
Up to 5		1.2 (0.90-2.43)
6-10	-	2.8** (1.83-5.60)
11 and more		4.6*** (2.56-10.47)

OR= odds ratio, CI= confidence interval, Chi-square statistic significant at

* ** P<0.001,

* * P< 0.01,

* P<0.05.

**Table 3:** Factors related to physical violence using logistic regression

Variable	OR (95% CI)	P-value
**Woman’s age**		
15-25	3.08* (1.26-5.35)	0.013
26-35	2.41* (1.06-5.50)	0.035
36-45	1.05 (0.86-4.73)	0.105
≥ 46	Reference
**Husband’s education(years)**		
0 to 5	2.28** (1.79-5.06)	0.001
6-12	2.0** (1.29-4.33)	0.007
>12 (college)	Reference
**Husbands’ occupation**		
Official employed	Reference
Self-employed	1.35 (0.74-2.46)	0.316
Manual skilled	2.93* (1.21-5.10)	0.016
semi-manual skilled(laborer)	3.62* (1.13-4.88)	0.030
**Husband’s smoking (≥25 cigarettes/day)**		
Yes	2.62** (1.51-4.52)	0.001
No	Reference	
**Husband’s drug abusing**		
Yes	2.1* (1.51-2.82)	0.02
No	Reference	

*Adjusting variables were composed of the woman’s age, the husbands’ age, the woman’s education, the husband’s education, the woman’s occupation, the husband’s occupation, housing tenure, duration of the marriage, and the husband’s cigarette smoking and drug abuse.

*** P<0.001,

** P<0.01,

* P<0.05.

### 3.3 Mental Violence

We also determined that 85.5% of the participants reported at least one or more events of any kind of mental violence. Participants were more likely to suffer from mild (46.5%) and moderate (35.6%) degrees of mental harassment, but 3.4% of them had experienced severe mental violence within the 12 months. Mental violence was described as any form of offensive words, shouting and wrath, derision and humiliation, preventing one’s wife from contact or visiting her parents or friends, physically locking up one’s wife at home, unplugging the phone at home, patriarchal control over the family earnings and household income, preventing the wife from working or getting an education, skepticism about one’s wife’s faithfulness, and threatening to divorce or re-marry by the husband.

Table 2 shows the association between having experienced mental violence by one’s husband and other background variables, such as age, educational level, occupation, housing tenure status, living in primary years after marriage, being a heavy smoker, and drug abuse by the spouse. For instance, housewives (P<0.05) and middle-aged women were found to have faced a significantly higher (P<0.01) proportion of mental violence by their husbands than employed and older women. Similarly, a significantly lower proportion of women who had higher education had experienced mental harassment compared to the situation in which both the woman and her husband had not completed their secondary education. In addition, a significantly lower percentage of women (P<0.001) whose husbands were official employees had experienced mental harassment compared to those whose husbands engaged in manual or semi-manual skilled work. As the years of marriage increased, women had a significantly higher (P<0.001) chance of being subjected to mental violence compared to those in the early years of marriage, and the highest prevalence rate was reported within the first 11-15 years of marriage, after which it decreased dramatically (P<0.001).

After adjusting for these variables, age and woman’s occupation were excluded from the model due to their strong correlation with the husband’s occupation and the duration of the marriage. However, low education level, heavy cigarette smoking, and drug abusage of spouse were significant factors associated with having experienced mental violence by the spouse. Logistic regression analysis indicated a risk reduction in mental violence risk among women whose spouse had completed their secondary education; in other words, achieving a higher educational level by the husband was associated significantly with decreased mental harassment when compared to situations in which the husband had not completed his primary education. Furthermore, the most consistent protective effect against mental violence against woman was observed among women whose spouses were official employees than when husbands were engaged in a semi-skilled or non-manual skilled work (P < 0.001). Conversely, increasing the years of marriage was associated significantly with 2.8 times increased risk of mental harassment compared to the living within the primary years of marriage. Moreover, heavy smoking and drug abusage of the spouse remained significant as risk factors for mental violence against women. In the final model, those activities increased the risk to 2.3 and 3.4 times, respectively, compared to those did not report such activities (Table 4).

**Table 4:** Factors related to mental violence using logistic regression

Variable	OR (95% CI)	P-value
**Husbands’ education**		
0 to 5	3.06** (1.14 -4.32)	0.001
6 to 12	2.7* (1.30-5.91)	0.03
> 12(college)	0.2** (-0.78-2.16)	0.008
**Husband’s occupation**		
Official employee	0.4*** (-0.14- 2.99)	0.001
Self-employee	1.2 (0.8-3.06)	0.82
Manual skilled	3.5*** (1.35-3.45)	0.001
Semi-manual skilled	3.8. *** (1.68-4.90)	0.001
**Duration of marriage(years)**		
Up to 5	Reference
6-10	1.6* (1.2- 3.22)	0.03
11 and more	2.8 ** (1.36- 4.20)	0.001
**Husband’s smoking(≥25 cigarette/day)**		
Yes	2.3** (1.76-3.96) Reference
No	0.001
**Husband’s drug abuse**		0.001
Yes	3.4*** (1.8-5.06)
No	Reference

Adjusting variables were composed of the woman’s age, the husband’s age, the woman’s education, the husband’s education, the woman’s occupation, the husband’s occupation, housing tenure, duration of the marriage, and the husband’s cigarette smoking and drug abuse.

*** P<0.001,

** P<0.01,

* P<0.05

We did not find any association between a history of any kind of reported childhood violence in couples, gender of the children, age gap with spouse, living with relatives (extended family), consanguinity marriage, grade of marriage, and living with children from a previous marriage with mental violence.

### 3.4 Collative Behaviors

At the end of the questionnaire, we assessed collative or adaptive behaviors of the victims who experienced any kind of IPV within the past 12 months. The findings indicated that more than half of the women (54.7%) had tried to negotiate with their spouses to resolve problems. Others preferred to give up and abandon the arguments (39.5%), to consult with family members (16.5%) or friends (11.8%), to engage in mutual fighting (15.4%), to leave home (1%), and to file a complaint in court (1%). Higher-educated couples and employed and older women (>35 years) tended to show more mutual negotiation compared to housewives, lower-educated women, and younger women (P<0.001). However, housewives tended to abandon the arguments with their spouses significantly more than employed women (P<0.05). Furthermore, mutual fighting and consultation with family or friends were reported significantly more often by younger and lower-educated women than others (P<0.001).

## 4. Discussion

Our findings showed that, although the reported IPV prevalence rate was lower than some internal and foreign studies (Agha Khani, Aghabiglooie, & Chehresaz, 2002; Bhuiya, Sharmin, & Hanifi, 2003; Diop-Sidibe, Campbell, & Becker, 2006; Farrokh, Eslamloo, & Booshehri, 2007; Koenig et al., 2003; Clark et al., 2009), domestic violence, especially mental violence, was widespread in Shahroud. In spite of the wide range of partner abuse (15% in Ethiopia to 71% in Japan) based on WHO’s Multi-country Study at 14 sites (Abramsky, et al., 2011), methods and types of physical violence are different at different sites. For instance, in Bangladesh, it was described as beating with the hands or wood or kicking (Bhuiya, Sharmin, & Hanifi, 2003), while 98% of the women in our study and in similar research in Iran described it as pushing, shaking, having objects thrown at them, or being slapped (Malek et al., 2004; Farrokh, Eslamloo, & Booshehri, 2007; Hashemi, 2011). Although the prevalence and types of IPV vary in different regions, there may be under-reported data that are affected by the methods of data collection, privacy, and the context of the interviews (as was done in our case when the participants were illiterate) and cultural norms that influence the estimation of the precise prevalence rates and types of IPV across the world. In communities, such as our Iranian society in which physical violence is considered a social crime and has legal fines, the prevalence of physical abuse may decline; conversely, the possibility of mental harassment may increase. Studies has shown that long-term mental violence by a partner is associated with negative health outcomes, such as depressive symptoms, chronic disease, and chronic mental illness (Naeem et al., 2008), and when both kinds of IPV are included in the logistic models, higher mental abuse rates were more strongly associated with these health outcomes than were physical abuse rates (Do et al., 2013).

The results of our study showed that educated and older women were less likely to experience physical violence by their spouses, and this was in agreement with the conclusions in similar studies conducted in Urmia (Arefi, 2003) and Ardabil (Narimani & Aghamohammadian Sherbaaf, 2005), two northwestern provinces in Iran. In addition, the protective effect of education against physical and mental violence appeared better when both the woman and her spouse had completed secondary education. Results from studies confirm such findings and explain the necessity of both females and males having access to educational attainment according to the Millennium Development Goals, especially in developing areas (Ahmad et al., 2007; DaFonseca et al., 2010; Diop-Sidibe et al., 2006). Presumably, young women who are usually less mature to handle marital relationships may also be economically vulnerable and at risk of submitting to man abuse (Shamu et al., 2011). It is believed that the more educational experience that girls attain, the better they are prepared for coping with the challenges of life. In addition, low-educated young women often lack enough experience in marital life and are more emotionally and financially dependent to their husbands in most of situations, which makes them more likely prone to experience IPV (Naeem et al., 2008).

Low socioeconomic status was associated with greater risk of physical and mental violence in our study as it has been at other sites across the world (Abramsky et al., 2011). Sociology literature indicate that the more resources are accessible for people, the more people’s capabilities are getting stronger by using power. Consequently, people who lack resources in the life, such as the financial or occupational resources, perceive no other source of power and are more likely to resort to violence; in other words, violence is considered as an ultimate resource, because it is used when other resources are not enough to achieve goals (Guruge, 2012). Another probable reason could be that the stress of handling the family expenses and the perception of occupational instability in most of manual and semi-manual skilled works may increase the risk of violence being perpetrated against wives. It has been stated that men living in poverty are unable to fulfill social expectations and the traditional role as a “successful man,” so violent acts towards women are the result (Jewkes, 2002).

In families in which higher-educated women are involved in some form of occupation in which they earn money, the women often have more power in financial decision making and have greater autonomy in family affairs compared to housewives, and they are less likely to be prone to IPV by their spouses (Nojomi, Agaee, & Eslami, 2007). In the present study, the occupational status of women was associated with risk of IPV before making adjustments for other variables (Table 2), which may confound or mediate the effect of this variable on IPV risk. However, our findings did not prove that such a difference exists in this domain. Alternatively, it seems that, in societies in which women’s status is in transition from low to high, as is the case in Iran, the risk of IPV tends to increase, because women have become aware of their own rights and seek enough power to challenge the authority of men (Naeem et al., 2008).

There also was an association between mental violence and the duration of marriage, as increasing the years of marrige increased the risk of mental violence against women, and this effect remained consistent after adjustment for other variables. However, there were some indications that women in newer relationships (less than five years) were at increased risk of IPV, compared to longer relationships (Abramsky et al., 2011). This result was unlike findings observed for physical violence, as increased risk of physical violence in the past year was associated with the younger age of women in the primary years after marrige. An alternate interpretation for our finding may be that the burden of financial problems and elder children’s needs increase along with their growing up, and the expectaion that these burdens should be met by the parents can lead to challenges between couples. Another reason that may account for the reduced inclination of husbands to commit physically violent acts toward their wives could be the presence of older children, which is not the case for younger couples.

Our study illustrates that women whose spouses were heavy smokers (≥25 cigarettes per day) and/or drug abusers were more likely to experience physiscal and/or mental violence than those whose spouse were not smokers or drug abusers. This finding is similar to those of other studies, which have found drug abuse to be a major cause of IPV (Hashemi et al., 2011; Taillieu & Brownridge, 2010; Ellsberg et al., 2008; Nojomi et al., 2007). Drugs often reduce inhibitions and lead to irresponsible behaviour such as violence and drug abusers are more likely to smoke many more cigarettes per day than those who do not use drugs. However, after controlling for drug abuse, the effect of heavy cigarette smoking on IPV was consistently significant.

Compared to the results from a few studies, there was no significat association between other background variables and the risk of IPV. For instance, in related studies, a history of childhood violence, the number and gender of children (especially having a daughter instead of a son), and living in a nuclear family have been shown to be risk factors of violence toward women (Malek Afzali et al., 2004; Arefi, 2003; Naeem et al., 2008). However, our findigs did not confirm these findings. The most likely explanation for these findings is that more than half of the respondents and their husbands had secondary and college educations and more than one-third of women were involved in an occupation in which they earned money (except for workers). It seemed that, when women had greater influence in the family and outside the home, they gained self confidence and increased their more capabilty to deal with community activities. For women, power can be aquired from several sources, such as education, making money, and having a role in community activities; an appropriate social situation and the attainment of wealth often are associated with low levels of violence for both women and men. Since low socio-economic status is strongly related to being abused, it is necessary to empower women by improving their educational level and raising their income to access and control the financial resources and consequently declining the chances of being abused (Shamu et al., 2011). In contrast, in families in which men dominate in economic issues and decisionmaking, the men are more likely to resort to violence to resolve disagreements with their wives, so a greater risk of partner violence exists in these situations (Naeem et al., 2008). Nonetheless, pre-disposing agents and risk factors of intimate partner violence vary even in similar settings in a country. Some of the differences may be explained by various factors, such as the design of the study, cultural differences between races, inclination of respondents to disclose violent experience, and women’s acceptance of interpersonal violence. On the othe hand, perception of violence and agreement to what behaviours are considered as partner violence are different among couples. So if a behavior is not seen as violent, it will be more acceptable by perpetrator and/or victim (Waltermaurer et al., 2013).

The most common adaptive behavior reported by more than half of respondents, particualrly highly-educated couples and older women, was mutual negotiation between spouses; giving up the argument was the next strategy to resolve the problems, and recourse to the legal system was hardly ever reported (1% of all participants). Results from other studies are similar to our findings that more than half of women who experienced physical or mental violence did not ask for assistance neither from family members nor legal athorities(Elsberg, 2006; Rabbani & Javadian, 2007; Garg & Singh, 2013). It often returns to the cultural norms and the extent of social and legal support in the community. Adequate social support was shown to be protective against IPV, and, in societies in which women are valued in their own right and are respected in the family and outside the home, they are less likely to be abused. Conversely, in settings in which male control is widely accepted, men use violence as a learned social behavior, and women learn to tolerate aggressive behavior (Bell et al., 2013). The most likely explanation for the concillatory behaviors among lower-educated women and housewives is that having a higher education and having access to finanicial resources empower women to challenge gender inequality through a rational path, such as mutual negotiation. In situations in which women do not have adequate social and economic support, they are more likely to return to their families, seek help from friends, or tolerate the living conditions; depending on the social traditions, they often have to be the first to concede to avoid continuing the argument in order to fulfill their role of ensuring ‘a good womanhood.’

This study has several limitations. First, respondents were recruited from accessible sites, and women who were considered to be out of reach were not included in the invesigation. Since abusive men are often restrict their wife’s movements and contacts with others, abused women are more likely to be isolated. Second, the measures of IPV relied on self-reporting by participants, and the prevalence rate and/or some of the background variables could have affected by the respondents’ lack of inclination to disclose the violence. Third, the reported previous history of childhood violence may be influenced by biased recall by women who have experienced partner violence (current violence) compared to never abused women. Finally, since the study had a cross-sectional design, causality between the variables cannot be concluded.

## 5. Conclusions

The findings of the present study indicate that a group of individual, household, and socioeconomic factors has a significant role in the occurrence of intimate partner violence. However, the importance and magnitude of some factors may vary across regions and for different races even in the same country. The salient effects of various factors, such as high educational levels and appropriate employment, which reduce poverty in society, highlight the need for access to higher education, especiaqlly for girls, and the need to change people’s attitudes towards gender norms. Due to the lack of a significant association of IPV with a number of variables, additional research is needed using different methods, such as qualitative or mixed-method studies. In addition, the study shuld be repeated in a less-developed area, such as a rural region, or with marginalized peole who are at greater risk of partner violence, such as refugees, divorced women, and homeless people. Finally, there needs to be routine screening and referral for IPV by care providers and more coordination between health sectors and criminal justice settings. In addition, Healthcare providers should understand more specifically about IPV and be aware of the community resources available to victims of the violence. They also should be skilled in detecting the risk factors of IPV, recognizing women who are at risk for partner violence, and providing appropriate healt services and guidance for women who are experiencing domestic violence.
